# Comparison of parathyroid hormone kinetics in endoscopic thyroidectomy via bilateral areola with open thyroidectomy

**DOI:** 10.1186/s12893-019-0656-8

**Published:** 2019-12-11

**Authors:** Daqi Zhang, Tie Wang, Gianlorenzo Dionigi, Jiao Zhang, Yishen Zhao, Gaofeng Xue, Nan Liang, Hui Sun

**Affiliations:** 1 Department of Thyroid Surgery, China-Japan Union Hospital Of Jilin University, Jilin Provincial Key Laboratory Of Surgical Translational Medicine, Jilin Provincial Precision Medicine Laboratory of Molecular Biology and Translational Medicine on Differentiated Thyroid Carcinoma, 126 Xiantai Blvd, Changchun city, Jilin province People’s Republic of China; 2 Division for Endocrine and Minimally Invasive Surgery, Department of Human Pathology in Adulthood and Childhood “G. Barresi”, University Hospital G. Martino, University of Messina, Via C. Valeria 1, 98125 Messina, Italy

**Keywords:** Thyroid surgery, Hypocalcemia, Intact PTH (iPTH), Central compartment lymph node dissection, Morbidity

## Abstract

**Background:**

In this study, we aimed to compare the kinetics of intact parathyroid hormone (iPTH) during the perioperative period of endoscopic thyroidectomy via bilateral areola approach (ETBAA) in the same period, following a traditional open thyroidectomy approach (OTA).

**Methods:**

We conducted a prospective observational study of patients who were undergoing thyroidectomy and level VI clearance. Patients who had been affected by papillary thyroid cancer (PTC) were stratified into three groups: those eligible for endoscopic treatment (ETBAA); patients who were eligible for ETBAA but had opted for OTA (OTA-L); and patients who were not suitable for endoscopic intervention (OTA-H). A process for locating parathyroid glands was utilized to stratify gland dissection laboriousness. In Type A, the gland is firmly fixed to thyroid gland. This type can be sub-classified into three subtypes. A1: the parathyroid gland is attached to the inherent thyroid capsule. A2: the gland is partially embedded in the thyroid gland. A3: the gland is located in the thyroid tissue. Type B is defined as a gland which is separated from the thyroid gland. The iPTH was sampled at wound closure.

**Results:**

There were 100 patients in each group. We found a significant difference between the ETBAA and OTA-H groups for type A2, as well as a loss of parathyroid glands and a number of parathyroid transplantation procedures. The endoscopic group was treated during an earlier stage of thyroid cancer. The iPTH profile of each group decreased, although this was the most consistent in the OTA-H group. A comparison of ETBAA with OTA-L demonstrates that the iPTH level change is similar.

**Conclusion:**

There is no advantage of endoscopic treatment for preserving parathyroid function.

## Background

The prevalence of hypocalcemia following thyroidectomy and central compartment lymph node dissection (CND) for papillary thyroid cancer (PTC) is high, with overall rates of 25% transient and 3% permanent [1]. This may be a result of manipulation of the parathyroid gland or devascularization, or may be caused by inadvertent removal along with the thyroid specimen [2–7].

Biochemical studies of post-thyroidectomy patients have shown that intact parathyroid hormone (iPTH) sampling is a valid, early predictor of the postoperative parathyroid gland state [1]. Limited data is available for assessing the state of the parathyroid following CND in endoscopic thyroidectomy [1–11].

This study aims to compare the kinetics of iPTH in the perioperative period of endoscopic thyroidectomy via bilateral areola approach (ETBAA) with the same period following a traditional open thyroidectomy approach (OTA).

## Methods

### Study design

Patients who had undergone total thyroidectomy and CND for thyroid cancer with postoperative iPTH evaluation were prospectively observed between October 2013 and April 2018. All participants gave informed consent before research commenced. The study was conducted in accordance with the Declaration of Helsinki, while the protocol was approved by the ethics committee of China-Japan Union Hospital of Jilin University (see protocol n. 2012-wjw-004).

### Setting

Research took place at the Academic Hospital, which is a single institutional, tertiary referral center.

### Participants

**Fig. 1:**
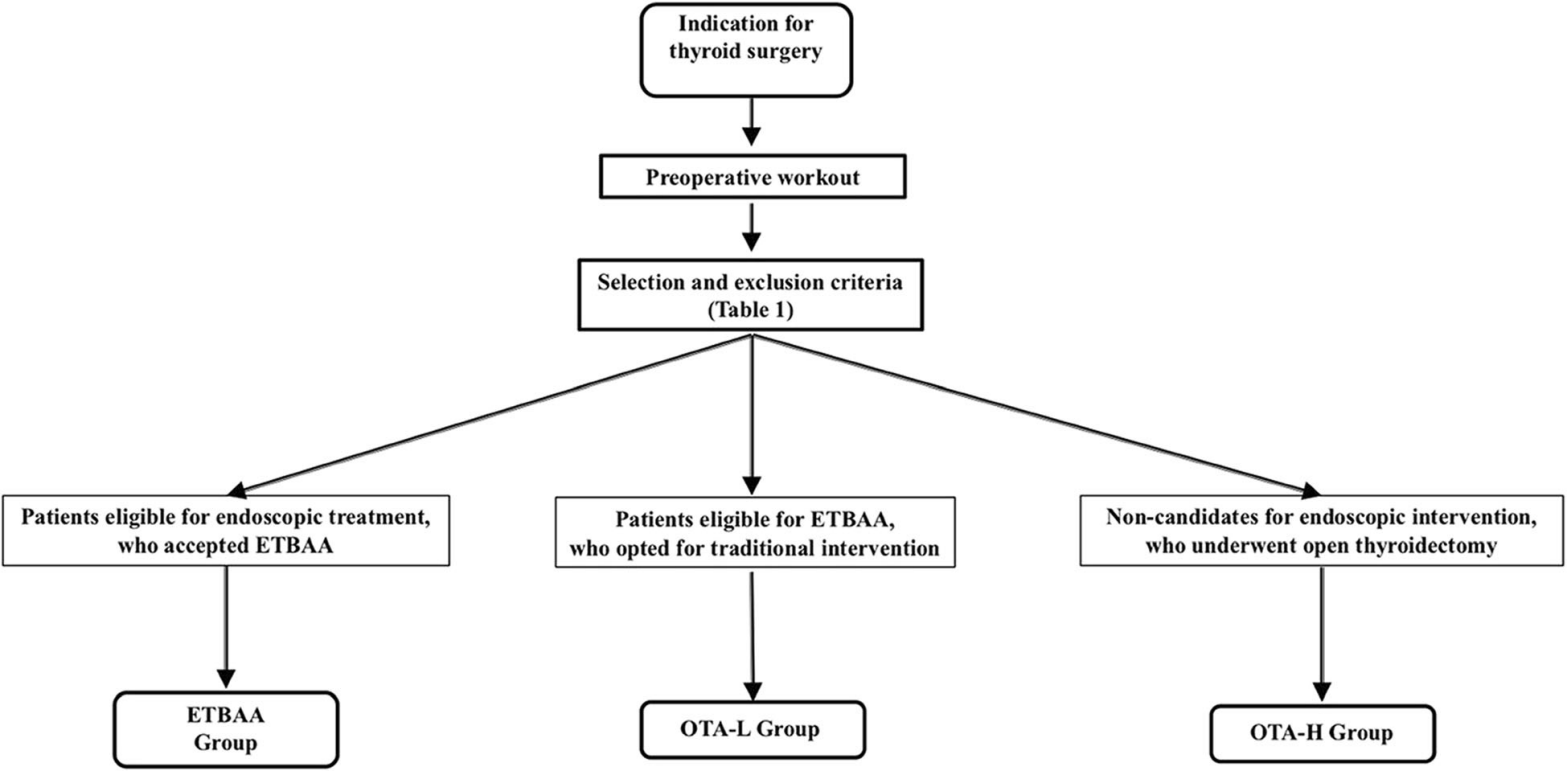
Flow chart for subgroup analysis. Patients eligible for endoscopic treatment, who accepted ETBAA were confined in Group ETBAA; patients eligible for ETBAA, who opted for traditional intervention in Group OTA-L; non-candidates for endoscopic intervention, who underwent open thyroidectomy were included in Group OTA-H

Patients who had undergone thyroidectomy without CND, concomitant parathyroid disease, renal failure, unilateral lobectomy, ‘berry picking’ dissection, re-done central neck dissections, high or low basal iPTH values (normal range 15-65 pg/ml) were excluded from analysis. Patients with vitamin D deficiency or preoperative calcium supplementation were also excluded. Inclusion and exclusion criteria for ETBAA can be seen in Table 1. The study only included patients with a malignant thyroid disease. Cancer patients were placed into three groups: (A) patients who were eligible for endoscopic treatment and who accepted ETBAA (ETBAA); (B) patients eligible for ETBAA yet who opted for OTA (OTA-L); (C) those cancer patients who were ineligible for endoscopic intervention who had undergone OTA (OTA-H). The inclusion criterion was cN0 on clinical and US examination for the ETBAA and OTA-L group, while those from the OTA-H group were ineligible for endoscopic intervention, including cN1. See Fig. 1 for a grouping flow chart. Patients could be included if they had PTC, been diagnosed preoperatively using cytological diagnosis, were diagnosed with N0 neck either clinically or following ultrasonography (US), and those whose diagnosis had been confirmed following intraoperative inspection.

**Table 1:** Detailed inclusion and exclusion criteria for ETBAA

Selection and Exclusion criteria	
Selection criteria	
Papillary thyroid cancer with low-risk factors^a^	
Dominant benign nodule with a diameter < 5 cm, whereas cystic nodule could be 6 cm or greater	
The patients needed a cosmetic requirement	
Exclusion criteria	
General factors	
Obesity and stocky neck	
Clinical history of radiation or surgery on the neck or chest	
Preoperative dysfunction of voice cord	
Thyroid-related factors	
Advanced cancer	
Local invasion	
Posteriorly located lesions	
Diffuse or adhesion or fixation enlargement of lymph node	
Evidence available of local or distant metastases	
Graves’ disease	
Severe thyroiditis	
Associated parathyroid disease	

^a^Low risk factors including lesion size < 4 cm, age < 55 years, no prior radiation, no distant metastases, no lymph node metastases, no extrathyroidal extension, no aggressive variant, and no first-degree family history of thyroid carcinoma

### Definitions and interventions

All participants were subjected to preoperative high-resolution US [12]. The 2009 Bethesda classification system of thyroid cytopathology was utilized [12]. Non-contrast slides from CT scans were frequently utilized during the initial evaluation of a solitary thyroid cancer.

### Surgical technique

The ETBAA technique has been previously described (Additional file 1: Video S1) [13]. The third and fourth intercostal incisions of male ETBAA patients are conventionally used for the endoscopic access. The middle incision is 12 mm, and the bilateral incisions are approximately 5–6 mm. The central neck dissection (level VI) is then performed similarly to that of women patients. Standardized neural monitoring was implemented in all groups [13]. All surgeries were performed by the same endocrine surgeon (DZ). Dissection and hemostasis were achieved in OTA with a Focus Plus device, and were achieved in ETBAA by means of an ACE plus device (Johnson & Johnson Company, Cincinnati, OH, USA). Drainage tubes were attached from the chest incision.


**Additional file 1: Video S1.** The procedure of protection and autotransplantation of parathyroid gland in ETBAA and open thyroid surgery. The video show that the ETBAA and open thyroid surgery should be carefully operated to protect parathyroid gland and its blood supply in situ as far as possible. The parathyroid gland with poor blood supply should be transplanted into sternocleidomastoid muscle in time.


### Parathyroid gland scheme location and dissection

**Fig. 2:**
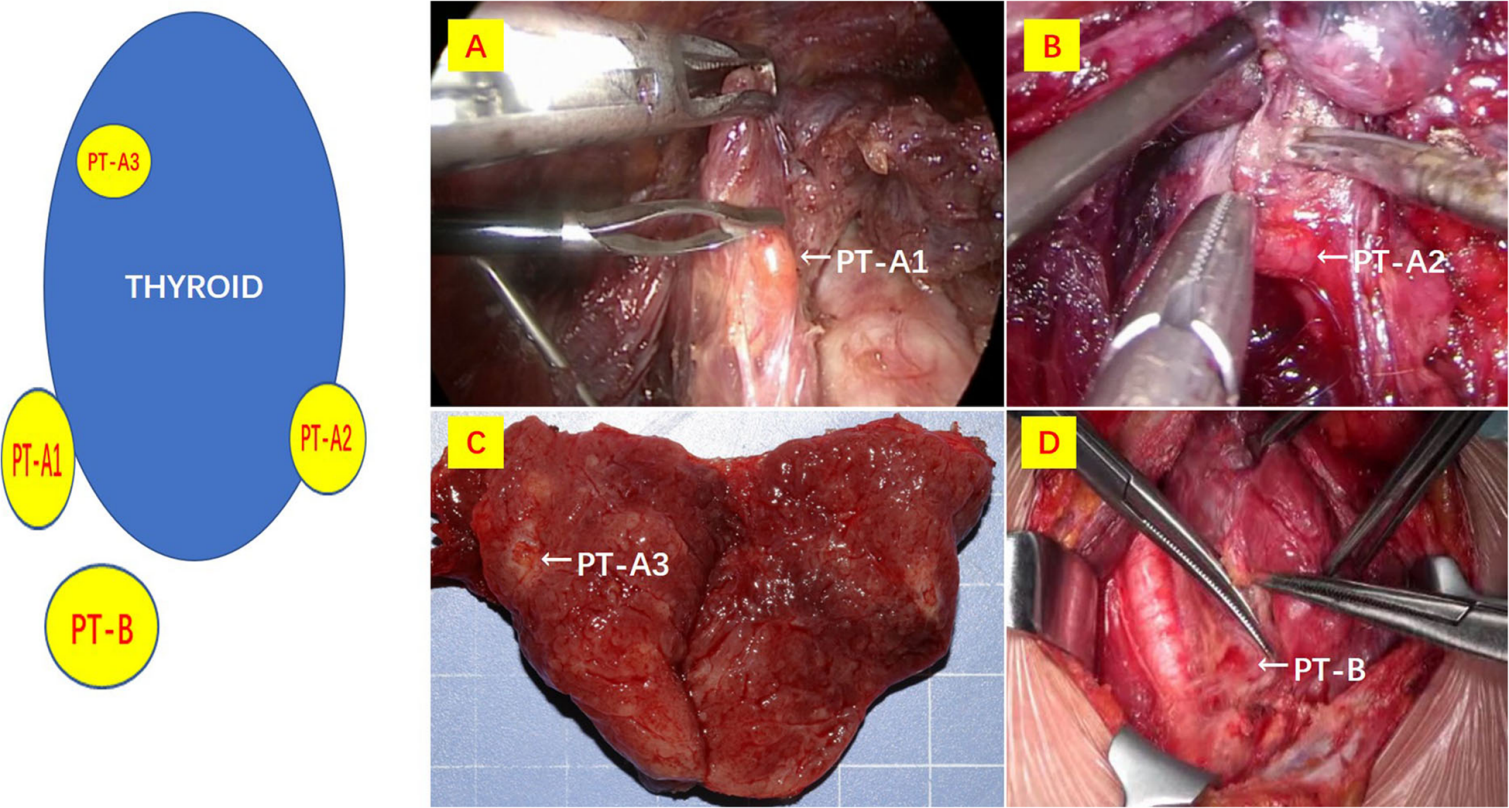
Design and intraoperative pictures of the dedicated localization scheme we used to describe the relationship between the parathyroid gland and the thyroid gland. Type A: parathyroid gland is tight, closely related to the thyroid gland. Type A is divided into three subtypes. **a** Type A1, parathyroid gland is attached to the surface of thyroid gland; **b** type A2, parathyroid gland partially or completely embedded in the thyroid gland, but located outside the inherent capsule of the thyroid; **c** type A3, the parathyroid gland is completely located in the thyroid tissue. **d** Type B is distant from the thyroid gland

Preservation of the parathyroid glands as well as their blood supply was attempted in all cases. Efforts to identify all four glands also took place in all cases. However, an extensive neck exploration to search for one or two missing glands was not carried out. A classification scheme for parathyroid glands location was utilized (see Fig. 2). Type A: the gland is firmly fixed to thyroid gland. Type A can be grouped into three subtypes. A1: the parathyroid gland is attached to the inherent thyroid capsule. A2: the gland is partially embedded in thyroid gland. A3: the gland is located in thyroid tissue and is discovered after thyroid gland excision. Type B can be defined as a gland which is separate from the thyroid gland [14].

### Parathyroid autotransplantation

In cases where the parathyroid glands had been damaged, auto-transplantation was undertaken. Prior to auto-transplantation, a section of a fragment of the parathyroid gland was frozen. Parathyroid tissue was stored in a saline solution after excision. Sliced parathyroid tissue and 1 ml of saline was withdrawn into a syringe and injected into the monolateral sternocleidomastoid muscle (Additional file 1: Video S1).

### Technique for CND

The techniques for CND applied have been described previously [8, 15–17].

### iPTH and calcium determinations

iPTH measurements were assessed by chemiluminescence (STAT-Intraoperative-Intact-PTH, Future Diagnostics, Wijchen, Netherlands). Venous samples were obtained before surgery and at skin closure. According to our protocol, if iPTH was < 15 pg/mL, then the subject received oral calcium and vitamin D substitution [15–17]. Serum calcium (range 2.2 to 2.6 mmol/L) was monitored at 24, 48, 72 h and + 15 days postoperatively. On follow-up visits at + 1, 3 and 6 months, both the serum calcium and the iPTH level were verified [15–17]. If two consecutive serum calcium determinations were within or above the normal value, then firstly calcium and secondly vitamin D were no longer given [15–17].

### Follow-up

Pre- and postoperative follow-up included laryngoscopy [15–17]. Measurement of serum Thyroglobulin, stimulated thyroglobulin hormone (TSH) and thyroglobulin antibodies were monitored in all patients after surgery in the follow up period [15–17]. After surgery, patients were given TSH-suppressive L-thyroxine therapy [12]. The seventh edition of the AJCC/TNM cancer staging system was used [12]. Papillary thyroid cancer indications for RAI treatment (4–5 weeks after surgery) include: > 45 years or < 18 years; tumor > 2 cm; thyroid capsule, soft tissue, adjacent organ tumor extension; LN+ (in any area of the neck); non-classical variants of PTC; M+; remnant ablation; BRAF+.

Neither profiles of thyroglobulin, TSH and thyroglobulin antibodies nor prevalence/dose of RAI analysis are the purpose of this preliminary report.

### Primary outcome

iPTH kinetics in ETBAA versus open surgery were considered as the primary end-point.

### Statistics

PTH < 15 pg/ml was utilized for statistical testing [15–17]. The cut-off value for the serum calcium level was 2.0 mmol/L [7, 15–17]. Analysis of the data was calculated using SPSS version 19.0 (SPSS Inc., Chicago, USA) and SAS version 9.4 (SAS Inc., North Carolina, USA). Data were presented as mean ± standard deviation. The differences between parathyroid gland outcomes, changes of PTH perioperative or serum calcium and calcium supplementation in the ETBAA, OTA-L and OTA-H groups were analyzed using Cochran’s and Mantel-Haenszel Chi-square test. The level of statistical significant was 0.05.

## Results

### Data collection analysis

**Fig. 3:**
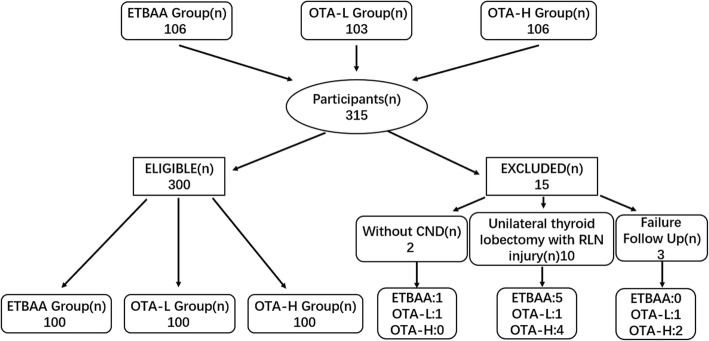
Flow diagram inclusion and intervention for the study

Over the six-year period of the study, 315 patients underwent total thyroidectomy and CND as a one-stage procedure. Figure 3 depicts a flow chart with the number of patients eligible and not eligible for the study. Based on the selection and exclusion criteria, 300 patients were eligible for the study. These were 44 men and 256 women, aged 16–54 years (median 37.7). Demographic, clinical, laboratory, operative, morbidity and histology data can been seen in Table 2. Mean length of follow-up was 20 ± 6 months (range five to 52 months). No instances of instrument malfunction (neuromonitoring, endoscopy, ultrasound scalpel and so on) were recorded in this study.

**Table 2:** Patients characteristics

	ETBAA (*N* = 100)	OTA-L (*N* = 100)	OTA-H (*N* = 100)
Age ± SD (years)	31.2 ± 9.31	36.2 ± 10.5	47.9 ± 15.7
Gender (M/F)	11/89	16/84	17/83
Central lymph node dissection			
Prophylactic	100	100	17
Therapeutic	0	0	83
Mean operating time (min)	124.8 ± 19.4	43.8 ± 9.29	57.9 ± 12.23
intraoperative blood loss (ml)	7.02 ± 3.44	6.56 ± 3.24	8.31 ± 3.15
Postoperative hospital stay time(D)	3.88 ± 0.52	3.13 ± 0.76	4.02 ± 0.98
Total drain volumes (ml)	63.34 ± 21.2	25.18 ± 18.3	52.28 ± 16.9
Mean draining days	1.85 ± 0.42	1.39 ± 0.37	1.89 ± 0.62
Parathyroid dysfunction (number)	7	6	15
Transient	7	5	12
Persistent	0	1	3
Hypocalcemia (number)	9	11	18
Transient	9	10	15
Persistent	0	1	3
Recurrent laryngeal injury (number)	7	2	6
Transient	7	2	4
Definitive	0	0	2
Histopathology			
papillary thyroid carcinoma	100	100	100
Thyroid volume (mm^3^)	7445 ± 438	7562 ± 492	8250 ± 509
Mean diameter of tumor (cm)	0.89 ± 0.52	0.93 ± 0.43	2.85 ± 1.83
Extracapsular invasion(+/total)	0/100	0/100	43/100
Parathyroid in specimen	4	3	7
Coexistence thyroiditis	9	11	22
Mean number of LN(n)	7.78 ± 2.03	2.99 ± 1.53	10.3 ± 3.4
Mean Metastatic (n)	2.17 ± 3.12	0.77 ± 1.29	3.47 ± 4.08

N is the number of patients in the different groups

Data, which fitted the normal distribution, were presented as Mean ± SD

### Comparative study

There were 100 patients in each group. In the ETBAA group, mean age was 31.2 ± 9.31, and there were 11 male and 89 female participants. In the OTA-L group, mean age was 36.2 ± 10.5, and there were 16 male and 84 female patients. In the OTA-H group, mean age was 47.9 ± 15.7, while there were 17 male and 83 female participants. The only significant difference between the ETBAA and OTA-L groups was the mean operating time and drainage (Table 2). The preoperative TSH (*P* = 0.17) and tireoglobulin levels (*P* = 0.26) were seen to be within the normal range for both groups. None of the patients in the study population had a preoperative recurrent laryngeal nerve (RLN) palsy. There was no significant difference found between the incidence of hypoparathyroidism (permanent 0% versus 1%; transient 7% versus 5%) and hypocalcemia (permanent 0% versus 1%; transient 9% versus 10%) (Table 2). A comparison of the ETBAA and OTA-H groups and of the OTA-L and OTA-H groups found that the mean tumor diameter, rate of extracapsular invasion and number of lymph node metastases were significantly different and were positively correlated with both hypoparathyroidism and hypocalcemia following surgery (Table 2). The endoscopic group were treated during an earlier stage of thyroid cancer. Regarding RLN injury, both transient and permanent unilateral palsy were found in the ETBAA group at 7% versus 0%, the OTA-H group at 4% versus 2% and the OTA-L group at 2% versus 0% (*p* > 0.05) (Table 2). No cases of bilateral palsy were found.

### Parathyroid gland identification, position and transplantation

**Fig. 4:**
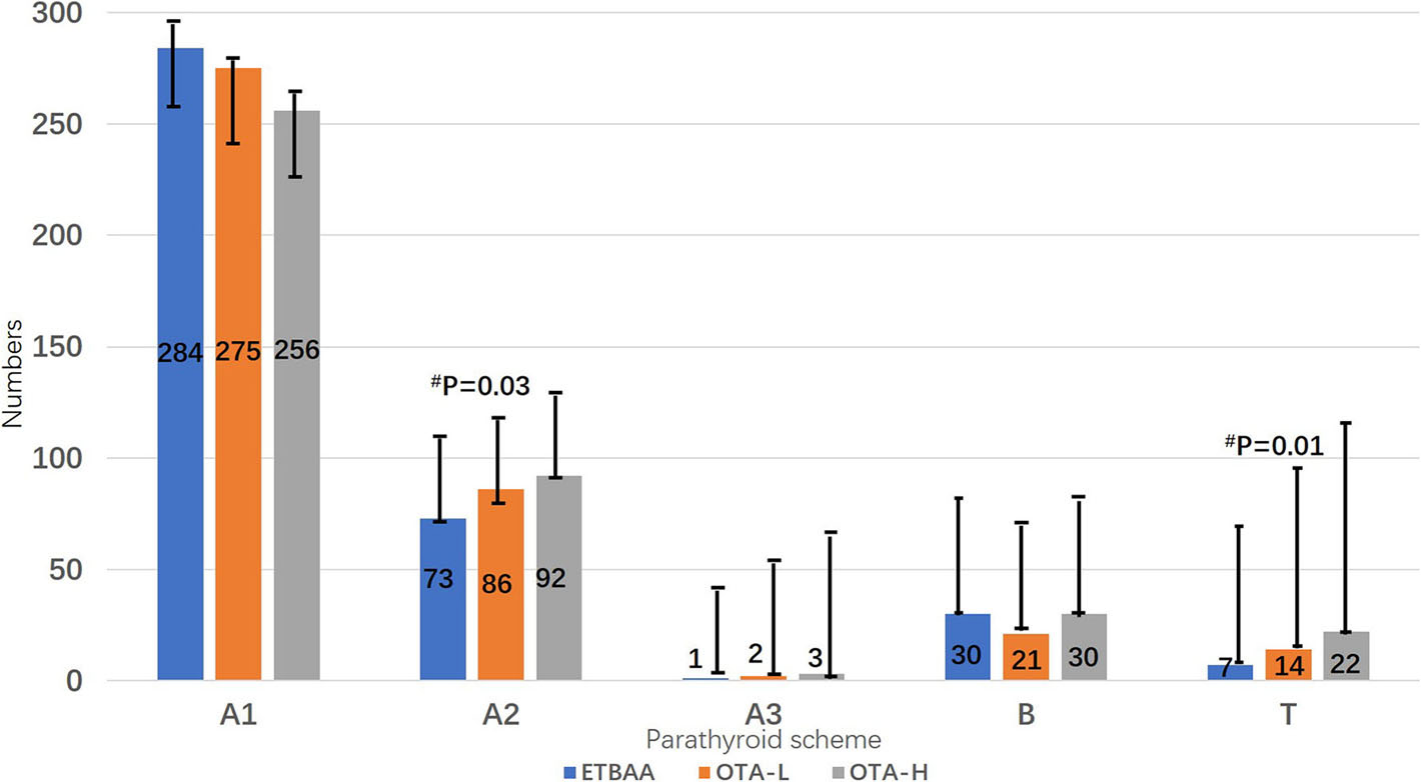
Comparative analysis for parathyroid location and strategy. A1, parathyroid gland is attached to the surface of thyroid gland; A2, parathyroid gland partially or completely embedded in the thyroid gland, but located outside the inherent capsule of the thyroid; A3, the parathyroid gland is completely located in the thyroid tissue. B is distant from the thyroid gland, which is easier to preserve in situ. T, Intraoperative auto-transplantation of parathyroid gland. ^#^P, Comparisons of groups between ETBAA and OTA-H

A significant difference was found between the ETBAA and OTA-H groups in parathyroid type A2, as well as a loss of parathyroid glands and a number of parathyroid transplantation procedures (*P* < 0.05). Details are described in Table 3 and Fig. 4.

**Table 3:** Parathyroid glands outcomes

	ETBAA	OTA-L	OTA-H
Number of glands identified			
1	0	0	1
2	3	4	5
3	6	8	6
4	91	88	88
Total	388	384	381
Location of glands identified			
Superior	200/200**	197/200	194/200 ^#^
Inferior	188/200	187/200	187/200
Right side	191/200	191/200	192/200
Left side	197/200*	193/200	189/200^#^
Superior right	100/100	99/100	98/100
Superior left	100/100*	98/100	96/100^#^
Inferior right	91/100	92/100	94/100
Inferior left	97/100	95/100	93/100
Parathyroid gland scheme			
Type A	358/388	363/384	351/381
A1	284/388	275/384	256/381
A2	73/388*	86/384	92/381^#^
A3	1/388	2/384	3/381
Type B	30/388	21/384	30/381
Incidental parathyroidectomy			
Location of removed			
Intrathyroidal	1/388	2/384	3/381
Subcapsular	6/388	9/384	12/381
Extracapsular	0/388**	3/384	7/381^#^
Number of removed			
1	7/388	9/384	15/381
2	0/388*	1/384	2/381^#^
3	0/388	1/384	1/381
Fragment	0/388	0/384	0/381
Auto-transplantation	7/388**	14/384	22/381^#^

*P < 0.05, **P < 0.01; analysis of the total three groups with Cochran’s and Mantel-Haenszel Chi-square (χ_CMH_^2^) test, while the variable of side was controlled

^#^*P* < α’, analysis of group ETBAA and group OTA-H with χCMH2 test, α’ = α/4 = 0.0125

### iPTH profile

**Fig. 5:**
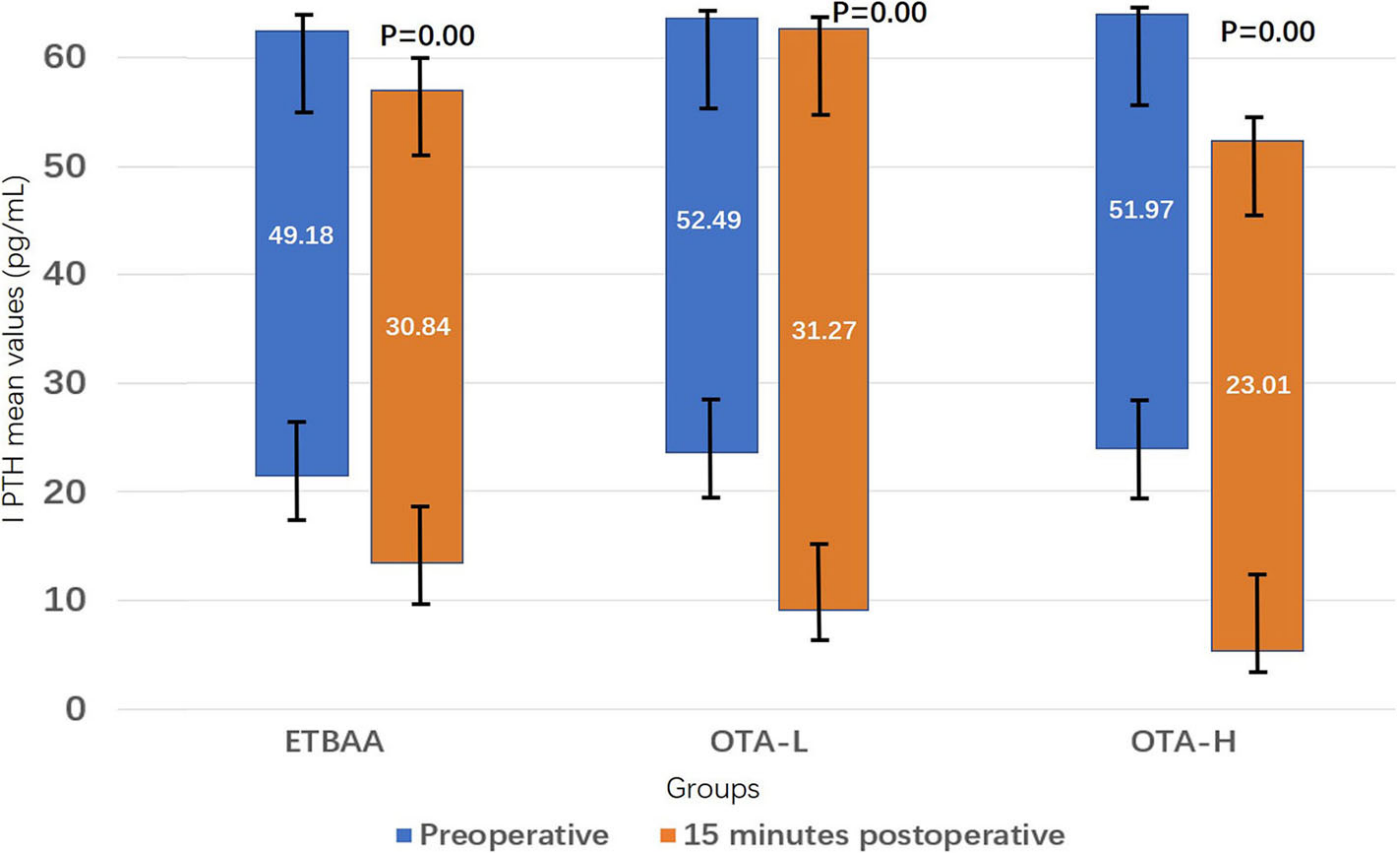
iPTH kinetics. P, Comparisons of Preoperative and 15 min postoperative in each group

No significant difference was found in preoperative iPTH values between the OTA-L, OTA-H and ETBAA groups (Table 4, Fig. 5). Fifteen minutes following surgery, the iPTH profile of each group decreased, although this was most consistent in the OTA-H group (Fig. 5). No significant difference was found in iPTH between the ETBAA and OTA-L groups. There was a significant difference found between the OTA-H and ETBAA groups, and between the OTA-H and OTA-L groups (Table 4). When comparing the ETBAA with OTA-L, the iPTH level change is similar.

**Table 4:** Changes of PTH before and after operation

	ETBAA	OTA-L	OTA-H
Preoperative (pg/mL)	49.18 ± 23.18	52.49 ± 20.34	51.97 ± 31
15 min postoperative (pg/mL)	30.84 ± 17.55^**^	31.27 ± 22.66^£^	23.01 ± 19.07^#^

*P < 0.05, **P < 0.01; analysis of the total three groups with Cochran’s and Mantel-Haenszel Chi-square (χ_CMH_^2^) test, while the variable of side was controlled

^#^*P* < α’, analysis of group ETBAA and group OTA-H with χ_CMH_^2^ test, α’ = α/4 = 0.0125

^£^*P* < α’, analysis of group OTA-L and group OTA-H with χ_CMH_^2^ test, α’ = α/4 = 0.0125

### Serum Ca and supplementation

**Fig. 6:**
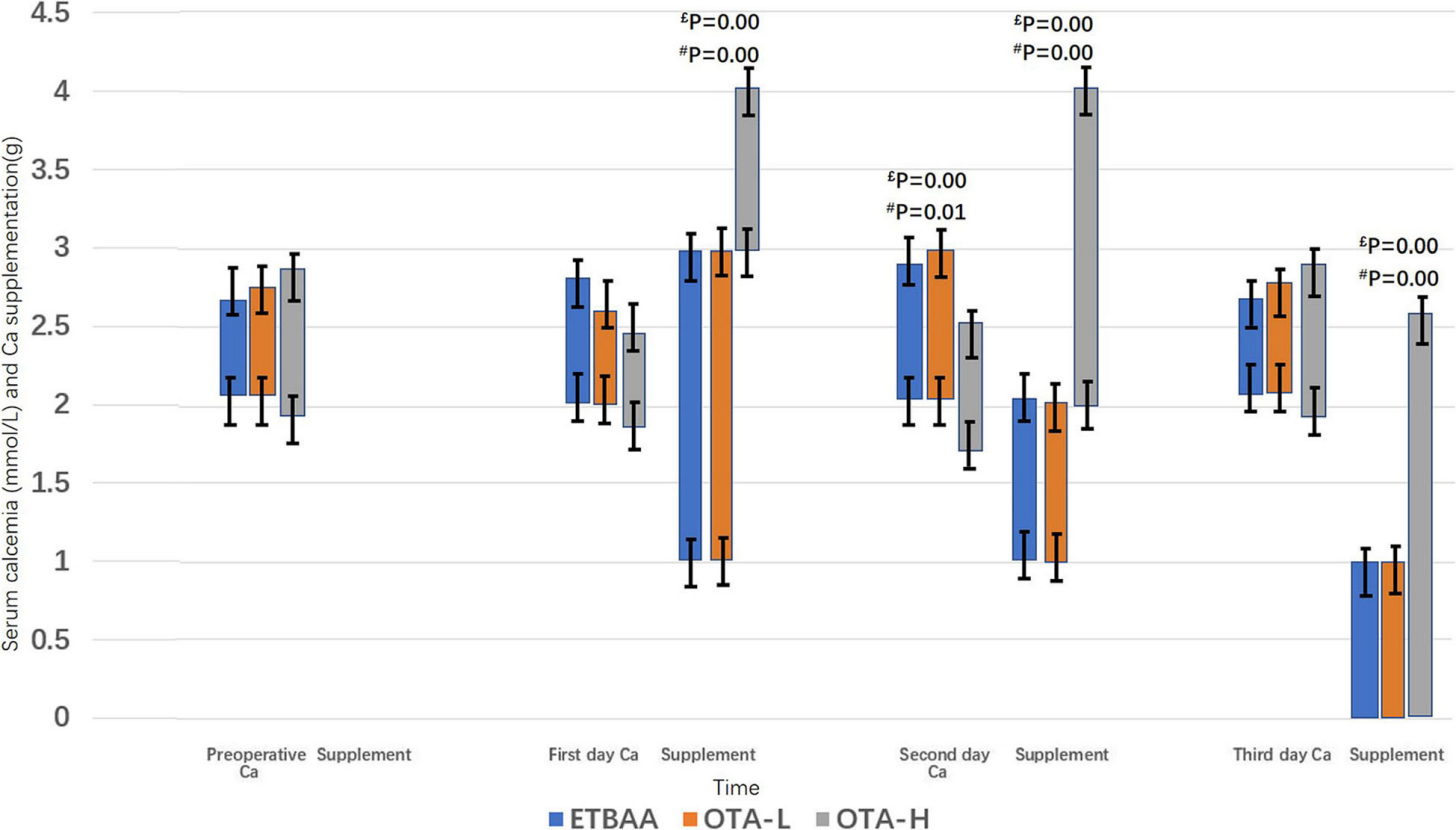
Serum Ca profile and supplementation. ^#^P, Comparisons of groups between ETBAA and OTA-H; £P, Comparisons of groups between OTA-L and OTA-H

No significant difference was found in basal serum Ca values for the OTA-L, OTA-H and ETBAA groups (Fig. 6, Table 5). On the first day following surgery, there was no significant difference found in serum Ca values between the OTA-L, OTA-H and ETBAA groups. No significant difference was found in calcium supplementation between the OTA-L and ETBAA groups.

**Table 5:** Serum calcium and calcium supplementation

	ETBAA	OTA-L	OTA-H
Preoperative			
Calcium (mmol/L)	2.32 ± 0.21	2.33 ± 0.19	2.29 ± 0.39
Supplementation (g)	0	0	0
First day postoperation			
Calcium (mmol/L)	2.28 ± 0.32	2.35 ± 0.22	2.13 ± 0.37
Supplementation (g)	2.56 ± 0.48^**^	2.59 ± 0.32^£^	3.82 ± 0.46^#^
second day postoperation			
Calcium (mmol/L)	2.21 ± 0.37^**^	2.27 ± 0.31^£^	2.09 ± 0.41^#^
Supplementation (g)	1.89 ± 0.32^**^	1.93 ± 0.26^£^	2.75 ± 0.41^#^
third day postoperation			
Calcium (mmol/L)	2.25 ± 0.18	2.26 ± 0.19	2.18 ± 0.39
Supplementation (g)	0.52 ± 0.61^**^	0.41 ± 0.61^£^	1.83 ± 0.37^#^

*P < 0.05, **P < 0.01; analysis of the total three groups with Cochran’s and Mantel-Haenszel Chi-square (χ_CMH_^2^) test, while the variable of side was controlled

^#^*P* < α’, analysis of group ETBAA and group OTA-H with χ_CMH_^2^ test, α’ = α/4 = 0.0125

^£^*P* < α’, analysis of group OTA-L and group OTA-H with χ_CMH_^2^ test, α’ = α/4 = 0.0125

On day two after surgery, no difference was found in the serum Ca values of the OTA-L and ETBAA groups. However, a significant difference was discovered in serum Ca values between the OTA-H and ETBAA groups, as well as the OTA-H and OTA-L groups. There was also a difference in calcium supplementation between OTA-H and ETBAA groups as well as the OTA-H and OTA-L groups.

On the third day after surgery, no significant difference was found in the serum Ca values between the OTA-L, OTA-H and ETBAA groups. Statistical analysis found a positive correlation in calcium supplementation between the OTA-H and ETBAA groups as well as between the OTA-H and OTA-L groups (Table 5, Fig. 6).

### Histopathology

Histopathology confirmed that all participants had suffered from PTC. There was a significant difference found in thyroid volume, mean diameter of tumor, extracapsular invasion, coexistence thyroiditis and level VI metastasis between the OTA-H and ETBAA groups as well as between the OTA-H and OTA-L groups (Table 2). A significant difference was found in the mean number of lymph nodes dissected between the OTA-L and ETBAA groups as well as the OTA-L and OTA-H groups (Table 2). Statistics found a positive correlation in the mean number of metastasis between the OTA-L and ETBAA groups as well as the OTA-L and OTA-H groups.

## Discussion

We described parathyroid gland-related kinetics in the context of the current practices of both OTA and ETBAA. In this prospective observational study, 300 patients who had undergone thyroidectomy and central neck dissection were reviewed. Patients were stratified into three groups: those eligible for endoscopic treatment (ETBAA); patients who were eligible for ETBAA and had opted for OTA (OTA-L); and patients who were not suitable for endoscopic intervention (OTA-H). iPTH measurements for each patient were taken at pre-established points during the periods before and after surgery.

The iPTH profile of each group decreased, although this was the most consistent in the OTA-H group. When comparing the ETBAA with OTA-L, the iPTH level change is similar. Therefore, we can conclude that there is no advantage to using endoscopic treatment to preserve parathyroid function.

Thyroidectomy and CND are starting to be included in international guidelines [8, 12]. There is, therefore, an increased interest in the precise description of morbidity related to the procedures. Parathyroid gland preservation is recommended during ETBAA, as are other endocrine endoscopic and open techniques [15–17]. The dearth of studies which address the kinetics of iPTH during ETBAA led us to investigate hormone profile in comparison to well-standardized procedures such as OTA. We hypothesized that differences in iPTH kinetics may result in different thyroid gland approaches [15–17]. This is the first study to analyze the iPTH profile in ETBAA.

No other studies have compared the kinetics of iPTH levels after thyroidectomy and CND in ETBAA versus open surgery. Our study clarifies the kinetics of iPTH following 300 thyroidectomy and lymph node clearance procedures. The results of this study suggest the relevance of perioperative iPTH decline in CND for both open and endoscopic procedures. We have described a decrease between preoperative iPTH levels which is significantly related to the number of lymph nodes dissected, their metastatic status, and the type of parathyroid gland location.

Fifteen minutes after surgery, the iPTH profile of each group had decreased, although this was most consistent in the OTA-H group. No significant difference was found in iPTH between the ETBAA and OTA-L groups following surgery. However, there was a significant difference between the OTA-H and ETBAA groups as well as between the OTA-H and OTA-L groups. Perhaps the endoscopic magnification of the parathyroid glands during ETBAA has the potential to enhance gland preservation [15–17].

There are limitations to the present study. It is important to identify factors that cause interpatient variability in iPTH responses. A significant difference was found between ETBAA and OTA-H groups in parathyroid type A2, as well as a loss of parathyroid glands and a number of parathyroid transplantation procedures (Table 3 and Fig. 4). The significant differences we found may also be due to the selection of participants. Patients who are eligible for endoscopic surgery have less extensive disease and improved general factors when compared to other patients (Table 1). This has the potential to contribute to a more difficult surgery in the open surgery group, which can in turn necessitate and lead to more extensive preparations during surgery, which then obviously increase the risk of damage to the parathyroid glands.

## Conclusion

Patients with PTC were stratified into three groups: those eligible for ETBAA; those who were eligible for ETBAA yet opted for OTA (OTA-L); and those who were not suitable for endoscopic approach (OTA-H). The postoperative iPTH profile of each group decreased, although this was most consistent in OTA-H group. When comparing ETBAA and OTA-L, iPTH level profiles were similar. Thus, there is no advantage of endoscopic treatment over open surgery for preserving parathyroid function.

## Data Availability

The datasets generated and/or analyzed during the current study are not publicly available due to protecting individual patient privacy but are available from the corresponding author on reasonable request.

## Abbreviations

CND: Central compartment lymph node dissection
ETBAA: Endoscopic thyroidectomy via bilateral areola approach
iPTH: Intact parathyroid hormone
OTA: Open thyroidectomy approach
OTA-H: Ineligible for endoscopic intervention who had undergone OTA
OTA-L: Patients eligible for ETBAA yet who opted for OTA
PTC: Papillary thyroid cancer
TSH: Stimulated thyroglobulin hormone
US: Ultrasonography
